# Structural determinants and distribution of phosphate specificity in ribonucleotide reductases

**DOI:** 10.1016/j.jbc.2021.101008

**Published:** 2021-07-24

**Authors:** Eugen Schell, Ghada Nouairia, Elisabeth Steiner, Niclas Weber, Daniel Lundin, Christoph Loderer

**Affiliations:** 1 Institute for Microbiology, Technische Universität Dresden, Dresden, Saxony, Germany; 2 Department of Biochemistry and Biophysics, Stockholm University, Stockholm, Sweden

**Keywords:** ribonucleotide reductases, phosphate specificity, enzyme catalysis, enzyme kinetics, nucleoside/nucleotide biosynthesis, nucleoside/nucleotide metabolism, nucleic acid enzymology, site-directed mutagenesis, GTDB, Genome Taxonomy Database, RNRs, ribonucleotide reductases

## Abstract

Ribonucleotide reductases (RNRs) catalyze the reduction of ribonucleotides to the corresponding deoxyribonucleotides, the building blocks of DNA. RNRs are specific for either ribonucleoside diphosphates or triphosphates as substrates. As far as is known, oxygen-dependent class I RNRs (NrdAB) all reduce ribonucleoside diphosphates, and oxygen-sensitive class III RNRs (NrdD) are all ribonucleoside triphosphate reducers, whereas the adenosylcobalamin-dependent class II (NrdJ) contains both ribonucleoside diphosphate and triphosphate reducers. However, it is unknown how this specificity is conveyed by the active site of the enzymes and how this feature developed in RNR evolution. By structural comparison of the active sites in different RNRs, we identified the apical loop of the phosphate-binding site as a potential structural determinant of substrate specificity. Grafting two residues from this loop from a diphosphate- to a triphosphate-specific RNR caused a change in preference from ribonucleoside triphosphate to diphosphate substrates in a class II model enzyme, confirming them as the structural determinants of phosphate specificity. The investigation of the phylogenetic distribution of this motif in class II RNRs yielded a likely monophyletic clade with the diphosphate-defining motif. This indicates a single evolutionary-split event early in NrdJ evolution in which diphosphate specificity developed from the earlier triphosphate specificity. For those interesting cases where organisms contain more than one nrdJ gene, we observed a preference for encoding enzymes with diverse phosphate specificities, suggesting that this varying phosphate specificity confers a selective advantage.

In many respects, ribonucleotide reductases (RNRs) are a thoroughly investigated class of enzymes. They catalyze the biologically crucial reduction of ribonucleotides to the corresponding deoxyribonucleotides, the building blocks of the genetic material all known life is based upon (1). Important features of this family of enzymes, such as the complex allosteric regulation of the substrate specificity or the generation mechanism of the catalytically active thiyl radical, have been studied extensively. However, one feature of RNRs is long known but still poorly understood. RNRs exhibit a specificity for the phosphorylation level of the ribonucleotide substrate (2). All enzymes characterized until today convert either ribonucleoside diphosphates or ribonucleoside triphosphates.

RNRs come in three different classes, which are largely defined by the radical generation mechanism of the enzyme (1). Oxygen-sensitive class III RNRs (NrdD) generate the radical *via* S-adenosyl-L-methionine (3, 4, 5), while class I RNRs (NrdAB) use the homolytic cleavage of molecular oxygen for radical generation (6, 7, 8). Class II RNRs (NrdJ) generate the radical by homolytic cleavage of 5′-deoxyadenosylcobalamin (cofactor B12) in an oxygen-independent manner (9, 10, 11). There is a loose correlation between RNR class and phosphate specificity. While NrdABs are nucleoside diphosphate reductases, NrdDs are nucleoside triphosphate reductases. In NrdJs, both diphosphate and triphosphate reductases have been observed (9, 11).

Since the first characterizations of NrdJ enzymes in the late 1960s, several enzymes from different organisms followed, yielding enzymes with both nucleoside diphosphate and triphosphate specificity (Table 1). One notable representative is the monomeric NrdJm from *Lactobacillus leichmannii,* which was used to investigate the reaction mechanism as well as the radical generation mechanism in B12-dependent RNRs (11). The enzyme reduces ribonucleoside triphosphates, and its three-dimensional structure was solved by X-ray crystallography (12). Another notable example is the dimeric enzyme from *Thermotoga maritima* with ribonucleoside diphosphate specificity (9). For this enzyme, several crystal structures were solved and used to give a detailed description of the allosteric regulation mechanism of the enzyme's substrate specificity (10, 13). For both enzymes, structures with substrate bound to the active site were solved, and the binding sites of the diphosphate or triphosphate moiety of the substrate were described (12, 13). For other NrdJ enzymes, the phosphate specificity was determined, but no structural information was obtained (Table 1).

**Table 1:** NrdJ phosphate specificity

Organism source	Specificity	Citation
*Rhizobium meliloti*	NDP	Inukai *et al*. (30)
*Thermotoga maritima*	NDP	Eliasson *et al*. (9)
*Thermoplasma acidophilum*	NDP	Eliasson *et al*. (9)
*Pyrococcus furiosus*	NDP	Fontecave (31)
*Stackebrandtia nassauensis*	NDP	Loderer *et al*. (17)
*Lactobacillus leichmannii*	NTP	Booker and Stubbe (32)
*Anabaena* sp.	NTP	Gleason and Olszewski (33)
Thermus virus P74-23	NTP	Loderer *et al*. (16)

NrdJ enzymes from different biological sources with characterized ribonucleoside phosphate specificity.

It is still unclear if the differing specificities serve a purpose and, if so, what this purpose could be (14). One hypothesis is that diphosphate specificity allows for a better regulation of the enzymes’ activity because ribonucleoside triphosphates are more abundant also in other metabolic pathways. The possibility of further regulation of dNTP synthesis might hence be the selective advantage that led to the evolution of diphosphate-reducing RNRs from a presumed triphosphate ancestor (2).

Another puzzle related to this question is the presence of two or more NrdJ enzymes in the same genome. Many microorganisms contain more than one RNR from different classes, but there are also genomes encoding genes of the same class. Considering the restrictions in terms of oxygen sensitivity (NrdD) and oxygen dependence (NrdAB), in some cases, the presence of several classes of RNRs can be explained by the need for dNTP synthesis under different oxygen regimes in facultative aerobic organisms (15). However, it is difficult to explain the co-occurrence of several oxygen-independent NrdJ enzymes in the same genome in this way, raising the question if phosphate specificity could be involved.

Thus, despite the large knowledge about RNRs and the crucial reaction they catalyze, little is known about the differing phosphate specificities. In this study, our objective was to gain insight into the basis of RNR phosphate specificity, by first defining and describing its structural determinants in NrdJ enzymes by means of mutational analysis. This knowledge was utilized to predict the distribution of the different phosphate specificities among all NrdJ enzymes. With this information at hand, we could study the phosphorylation levels exhibited by NrdJs in genomes encoding more than one copy of the enzyme.

## Results

**Figure 1:**
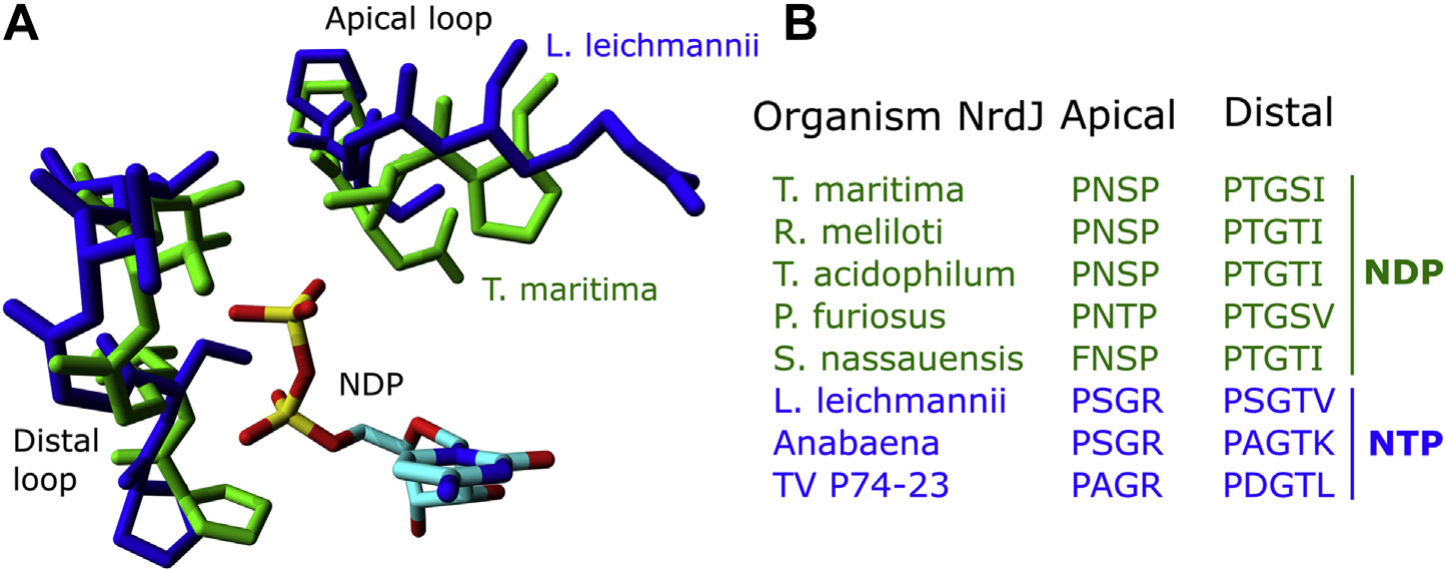
**RNR phosphate-binding site.***A*, structure of the phosphate-binding site in ribonucleoside diphosphate and triphosphate reductases. Diphosphate reductases are represented by the NrdJ from *Thermotoga maritima* (PDB ID: 1XJN, *green*) and triphosphate reductases by the NrdJm from *Lactobacillus leichmannii* (PDB ID: 1L1L, *blue*) (12, 13). *B*, sequence alignment of the apical and distal loop of the phosphate-binding site in enzymes with experimentally determined ribonucleoside triphosphate and diphosphate reductase activity. RNRs, ribonucleotide reductases.

We compared the structures of the triphosphate-reducing monomeric NrdJm from *L. leichmannii* and the diphosphate-reducing NrdJd from *T. maritima* (12, 13). In both enzymes, the phosphate-binding site consists of two loops (Fig. 1*A*). One loop, with an arrangement parallel to the phosphate groups of the substrate, forms the distal part of the binding pocket. A second loop defines the binding pocket in apical direction with respect to the phosphate groups. While the distal loops in both enzymes are very similar, the apical loop differs significantly in three positions. In the nucleoside triphosphate reductase from *L. leichmannii*, P-S-G-R form the apical loop, whereas it is P-N-S-P for the diphosphate reductase from *T. maritima*. In the latter one, the serine forms a hydrogen bond to the β-phosphate group of the diphosphate substrate. In the triphosphate reductase, a glycine at this position opens the space for the γ-phosphate group of the triphosphate substrate.

To test whether these amino acids may be involved in the phosphate specificity, the corresponding positions were investigated in other NrdJ enzymes (Fig. 1*B*). We found that, in diphosphate reductases, the sequence motif P-N-S-P is highly conserved among the characterized enzymes. Even with diphosphate-specific class I (NrdA) enzymes, this motif is present, although with more variability. For the three characterized NrdJ enzymes with triphosphate specificity, the G-R pair is conserved. The apical loop of the phosphate-binding site hence appears to be the structural determinant for the phosphate specificity in class II RNRs.

**Figure 2:**
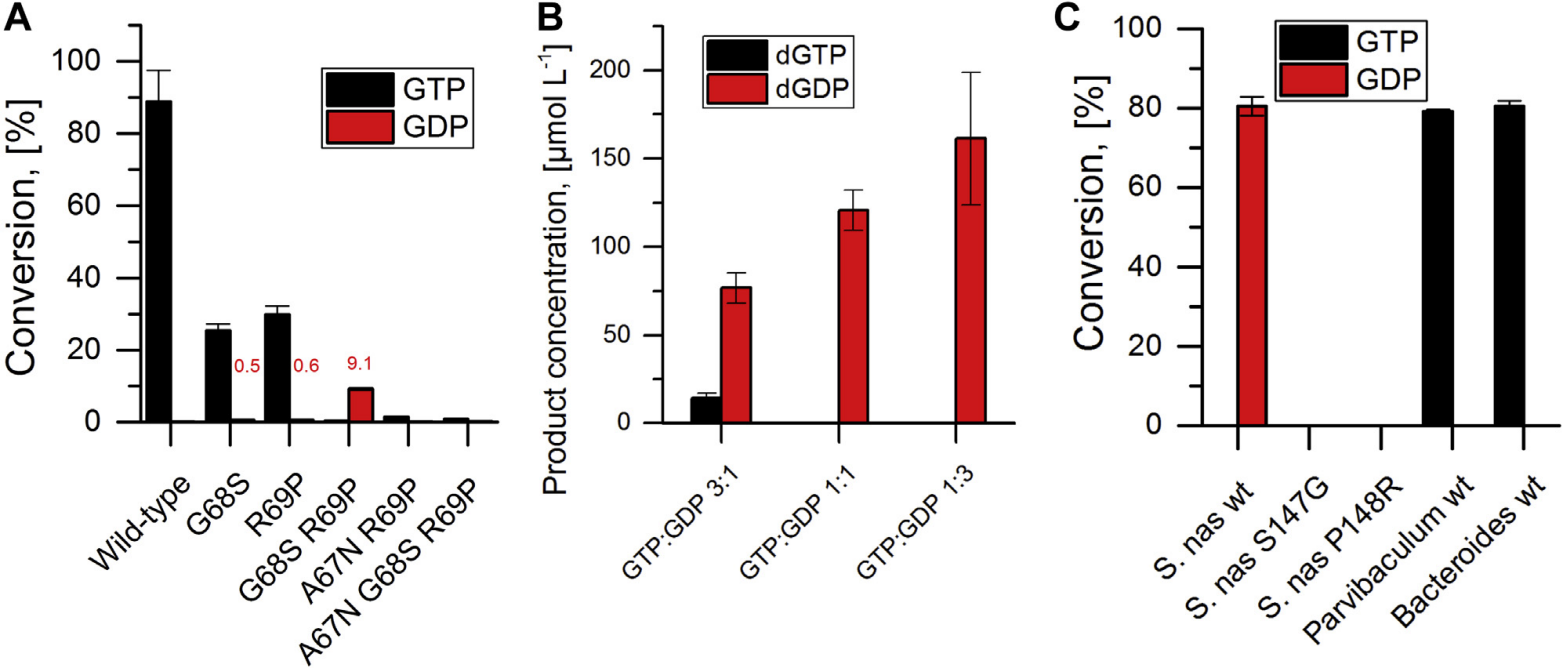
**Substrate test of ribonucleotide reductases.***A*, conversion of GDP and GTP to the corresponding deoxyribonucleotides by TVNrdJm WT and phosphate-binding site variants for guanosine diphosphate and triphosphate in the presence of the effector dTTP. Measurable conversions of GDP are stated in *red numbers* above the respective bar. *B*, conversion of GDP and GTP by TVNrdJm G68S R69P in a substrate competition experiment: The substrates GTP and GDP were applied in one pot with the following concentrations: 3:1 GTP 1.5 mmol L^−1^, GDP 0.5 mmol L^−1^; 1:1 GTP 1.0 mmol L^−1^, GDP 1.0 mmol L^−1^; 1:3 GTP 0.5 mmol L^−1^, GDP 1.5 mmol L^−1^. *C*, conversion of GDP and GTP to the corresponding deoxyribonucleotides by WT and phosphate-binding site variants for guanosine diphosphate and triphosphate in the presence of the effector dTTP. The error bars indicate the SD from three independent experiments. S. nas, *Stackebrandtia nassauensis.*

We put this hypothesis to the test first by grafting the apical loop of the diphosphate reductase (P-N-S-P) on a triphosphate reductase (P-S-G-R). For this experiment, we applied the thermostable triphosphate-specific NrdJm from the Thermus virus P74-23 (TVNrdJm) (16) as a recipient. The grafting was performed by creating the fully grafted triple mutant as well as several of the intermediate single and double mutants. The five variants as well as the WT enzyme were tested for the conversion of GDP/GTP in the presence of the effector dTTP. The variants TVNrdJm–G68S and TVNrdJm–R69P showed reduced conversion of the natural substrate GTP with traces of GDP reduction (Fig. 2*A*). The triple mutant TVNrdJm–A67N/G68S/R69P as well as the double mutant TVNrdJm–A67N/R69P showed no significant conversion of either GDP or GTP. Solely, the double mutant TVNrdJm–G68S/R69P showed conversion of GDP, 23-fold higher than GTP. A substrate competition experiment was performed with GDP and GTP to investigate the specificity of this variant (Fig. 2*B*). Applied in equal concentrations, GDP is converted with no detectable conversion of GTP. In a 3-fold excess, GTP is converted still with a 5.5-fold excess of dGDP production. In comparison with the activity of the WT enzyme on its natural substrate, the conversion of the variant under the given conditions was reduced by a factor of 10. Despite this loss of activity, the enzyme clearly changed the preference from a triphosphate to diphosphate substrate in response to the exchange of two amino acids in the apical loop of the phosphate-binding site.

To test if the exchange leads to a reversal of the phosphate specificity in the opposite direction, the grafting was performed from the triphosphate reductase G-R to the diphosphate reductase S-P in the diphosphate-specific NrdJd from *Stackebrandtia nassauensis* (SnasNrdJ) (17). The two single mutants SnasNrdJd–S147G and SnasNrdJd–P148R showed no activity on any of the substrates (Figs. 2*C* and S4). No soluble expression was obtained for the double mutant SnasNrdJd–S147G/P148R (Fig. S1).

**Figure 3:**
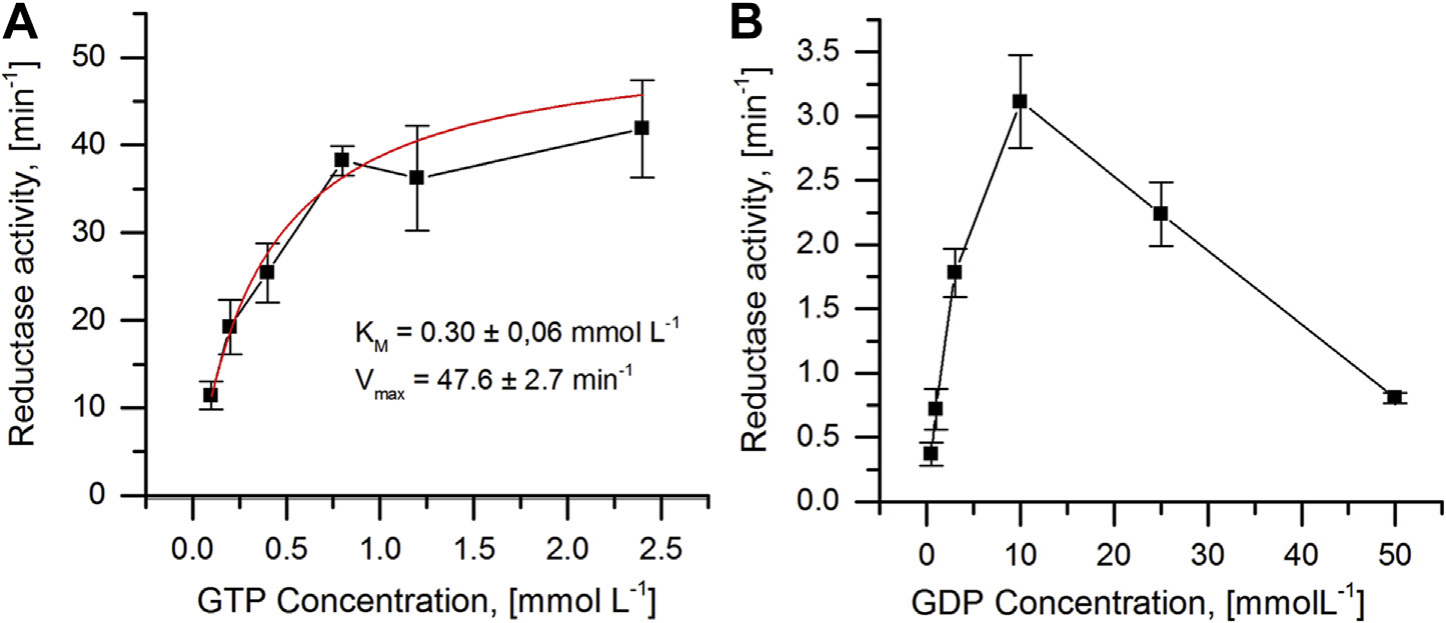
**Substrate affinity and maximal reaction velocity.** Dependence of substrate concentration and enzymatic activity for (*A*) the TVNrdJm WT and (*B*) the NrdJm–G68S/R69P double mutant for guanosine diphosphate and triphosphate, respectively. The fit to the Michaelis–Menten equation is shown as a *red line* in the graph. All experiments were conducted in the presence of the effector dTTP. The error bars indicate the SD from three independent experiments.

To further characterize the effect of the amino acid exchanges and to investigate the observed loss of activity, kinetic parameters were determined for the WT TVNrdJm enzyme and the TVNrdJm–G68S/R69P double mutant. The experiments were performed with guanosine nucleotides (GTP/GDP) in the presence of the effector dTTP. The WT enzyme had a *K*_M_ value of 0.30 ± 0.06 mmol L^−1^ with a *k*_cat_ of 47.6 ± 2.7 min^−1^ for the GTP substrate (Fig. 3*A*). The G68S/R69P double mutant did not show a simple Michaelis–Menten–like behavior, yielding decreasing enzyme activities at high GDP concentrations (Fig. 3*B*). A fit of the experimental data to the Michaelis–Menten model with substrate surplus inhibition did not converge within the tested data range. A calculation of apparent kinetic parameters was performed on a dataset with lower substrate concentrations, where the inhibition was not obvious yet. This yielded an apparent *K*_M_ value of 6.85 ± 1.94 mmol L^−1^ with a *k*_cat_ of 3.8 ± 0.4 min^−1^ (Fig. S5). Owing to the omission of inhibitory effects in this model, this value can only serve as the lower boundary of the actual *K*_M_ value of this enzyme for GDP. The highest measured activity of the TVNrdJm–G68S/R69P double mutant 3.1 min^−1^ at a substrate concentration of 10 mmol L^−1^ corresponds to 7% of the maximal activity of the WT enzyme for GTP.

**Figure 4:**
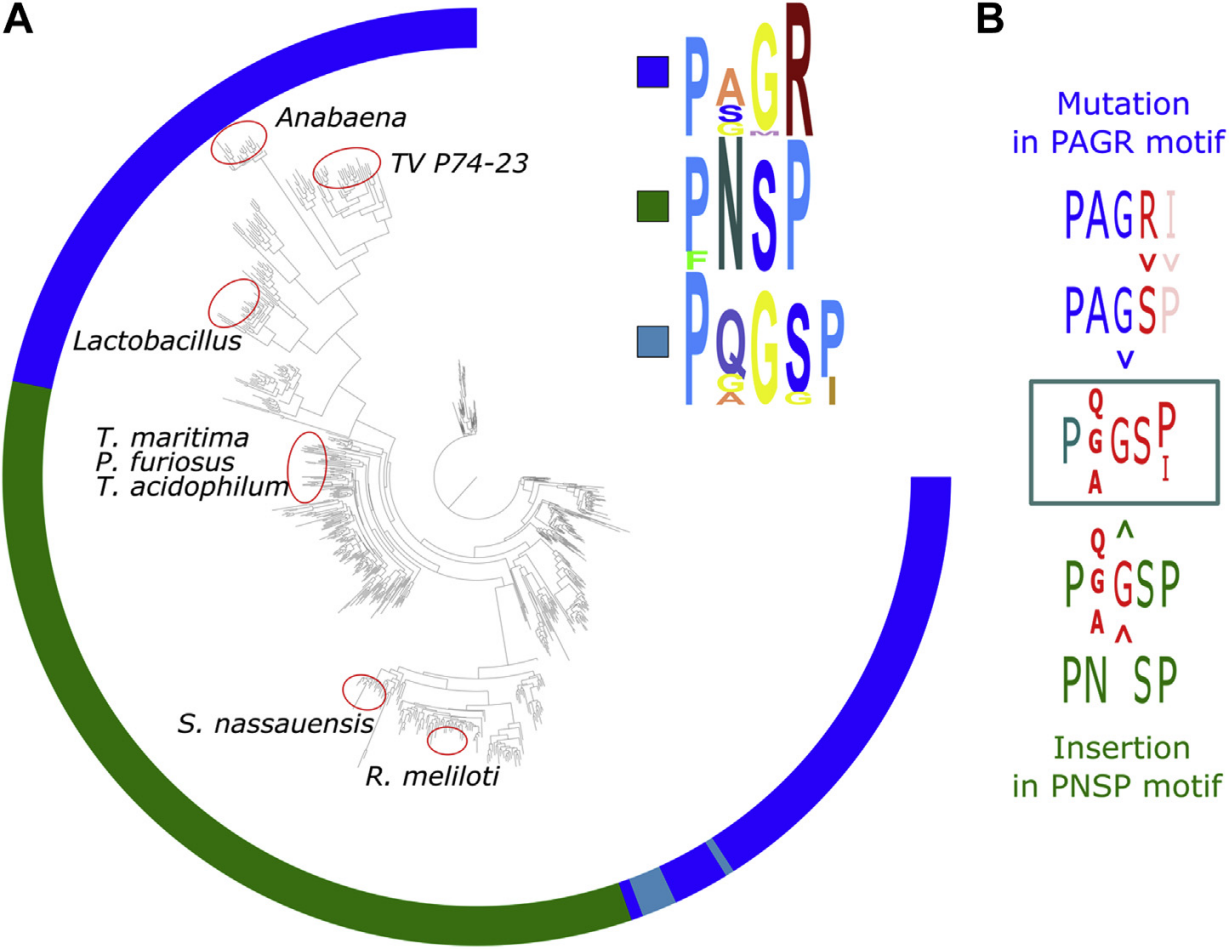
**Distribution of phosphate-binding site motifs in class II RNRs.***A*, the figure shows a phylogenetic tree of NrdJ (class II) RNRs. The *colored circle* around the tree shows the sequence motif of the apical loop in the phosphate-binding site in the respective RNR: P-A-G-R (*blue*), P-N-S-P (*green*), P-Q-G-S-P (*cyan*). The approximate locations of the NrdJ enzymes with known phosphate specificity from Table 1 are marked with *red ellipses*. *B*, the figure shows the possible connections between the P-Q-G-S-P (*cyan*) motif and the P-A-G-R (*blue*) and P-N-S-P (*green*) motifs, respectively. RNRs, ribonucleotide reductases.

Having identified the critical sequence motif, we proceeded with investigating its variability and abundance in the known variety of class II RNRs. To this end, we analyzed an alignment of a representative selection of 1655 NrdJ sequences, identified the positions that determine phosphate specificity, and mapped them on a phylogeny (Fig. 4*A*). More than 97% of the NrdJ sequences possess either the P-N-S-P (44%) or the P-A-G-R (53%) motifs, corresponding to diphosphate or triphosphate specificity, respectively. In the P-N-S-P motif, also a phenylalanine can occur at position 1 while the other three positions are fully conserved. In the P-A-G-R motif, the second position is less well conserved with serine and glycine residues occurring besides alanine (Fig. 4*A*). At position three, methionine occurs in the motif besides glycine, always in combination with a serine in position 2. This P-S-M-R motif represents 2.1% of all sequences. To test its phosphate specificity, we produced a TVNrdJm variant with the corresponding double mutation A67S/G68M. Despite successful soluble expression and purification of the variant, no activity could be observed (Fig. S6).

Another motif, appearing in 1.7% of the sequences, is P-Q-G-S-P. To test the phosphate specificity of this motif, we produced TVNrdJm variants with the corresponding mutations. For the P-Q-G-S-P motif, it is not clear, whether it originates from the P-N-S-P or the P-A-G-R motif. Thus, the motif could derive from P-N-S-P by a glycine insertion at position 3 and a mutation in position 2 or from mutations in the P-A-G-R motif (Fig. 4*B*). To account for both possibilities, two TVNrdJm variants were constructed, the TVNrdJm insertion 68G/G69S/R70P triple mutant (P-A-G-S-P) and the TVNrdJm–R69S (P-A-G-S). We decided to not exchange alanine in position 2 of the motif to glutamine because it also appears frequently in the original P-Q-G-S-P motif instead of the glutamine. Despite successful soluble expression and purification of the described variants, none of these enzyme variants was active on any of the tested substrates (Fig. S6).

Because the implementation of the two minor motifs in the TVNrdJm did not provide information about the respective phosphate specificity, we proceeded with the test of natural enzymes with the respective motifs. After a bioinformatical screening of the available sequences, we selected the NrdJ enzymes from *Parvibaculum* sp. and *Bacteroides* sp. UBA7333 for the P-S-M-R and the P-Q-G-S-P motif, respectively. Both enzymes are B12-dependent RNRs (Fig. S2) and show nucleoside triphosphate specificity (Fig. 2*C*).

The identification of the phosphate specificity–determining motif in the full variety of existing NrdJ sequences allows a prediction of the phosphate specificity. This can be utilized to tackle the question about the co-occurrence of NrdJ enzymes in various organisms. We investigated whether there exists any correlation between co-occurring NrdJs and their phosphate specificity. Of 1108 organisms with more than one NrdJ, 760 contained enzymes with differing predicted phosphate specificity. In contrast, there are only 113 organisms exclusively with enzymes with predicted triphosphate specificity and 235 exclusively with diphosphate specificity.

## Discussion

The grafting of a part of the apical loop from a ribonucleoside diphosphate reductase to the ribonucleoside triphosphate reductase from the Thermus virus P74-23 resulted in a change of selectivity toward diphosphate nucleosides. The high *K*_M_ value estimated for the NrdJm–G68S/R69P variant and the diphosphate substrate shows that the binding happens with comparably low affinity. The double mutant is hence by no means a perfect diphosphate reductase, and more mutations are likely necessary to reach the affinity of a true diphosphate-reducing enzyme. Still, the exchange of two amino acids was sufficient to introduce a preference for diphosphate substrates, showing the importance of this motif as a structural determinant of RNR phosphate specificity. Based on the available crystal structures of the two NrdJ enzymes from *L. leichmannii* and *T. maritima* (12, 13), we can propose a mechanism for the generation of the differing phosphate specificities. In diphosphate reductases, the shape of the apical loop is defined by a proline residue. The serine residue is oriented toward the substrate and forms a hydrogen bond to its β-phosphate group. This is not only stabilizing the interaction with the diphosphate substrate but also effectively locking out the triphosphate substrate through sterical hindrance. In triphosphate reductases, the presence of an arginine leads to a reorientation of the apical loop. In combination with the exchange of serine by glycine, the binding pocket is opened up for the sterically more demanding triphosphate substrate.

The alteration of the selectivity of the diphosphate reductase from *S. nassauensis* to reduce triphosphate substrates did not work in contrast to the change of Thermus virus P74-23 NrdJm in the opposite direction. However, this could be due to the chosen model enzyme itself. Mutations tend to decrease the stability of an enzyme (18) and the fact that the two tested enzymes have widely different thermostability might play a role. The more stable triphosphate reductase from a thermophilic source remained stable despite the mutations, while the less-stable diphosphate reductase from a mesophilic source was inactivated. On the other hand, it could be more difficult to open the binding pocket for the structurally more demanding triphosphate substrate than to close it. Thus, for the change from diphosphate to triphosphate specificity, more mutations in the second shell of the active site are conceivably necessary.

With the apical loop as determinant for the phosphate specificity in NrdJ enzymes, we investigated the distribution among known NrdJ sequences. The diphosphate reductases with the P-N-S-P motif formed a potentially monophyletic group within the triphosphate reductases, indicating a single origin of diphosphate specificity. Because class III RNRs (NrdD) as far as is known always reduce triphosphate substrates, the ancestral RNR, the “urRNR”, was likely a triphosphate reducer too (2). In contrast, class I RNRs (NrdA) that evolved from class II (NrdJ) are all diphosphate reducers, indicating that NrdA evolved from a diphosphate-reducing NrdJ. Phosphorylation level of the substrate hence appears to be an evolutionarily stable characteristic of RNRs, having changed probably only once in NrdJ. Possible exceptions were identified in the form of two motifs, P-Q-G-S-P and P-S-M-R, where the respective phosphate specificity was not clear. Because the implementation of the motifs in the Thermus virus NrdJm background yielded only inactive variants, we turned to NrdJ enzymes that naturally contain these motifs. The test of the enzymes from *Parvibaculum* sp. (P-Q-G-S-P) and *Bacteroides* sp. UBA7333 (P-S-M-R) yielded triphosphate specificity for both enzymes. Thus, both motifs are variants of the initial triphosphate motif that retained the triphosphate specificity.

In genomes which encode more than one nrdJ gene, we observed the co-occurrence of diphosphate and triphosphate reductases in 69% of all cases. This indicates a selective advantage for organisms carrying enzymes with differing phosphate specificities. This may explain why some organisms sustain two or more NrdJ enzymes, which at first glance seems redundant. The nature of the selective advantage however remains elusive and can only be answered in the light of the selective force that led from triphosphate to diphosphate reductases in the first place.

In this study, we identified the apical loop of the phosphate-binding site as the structural determinant for NrdJ phosphate specificity by mutational analysis. We used this knowledge to predict the distribution of this feature among the recent NrdJ enzymes and found a single major evolutionary split that gave rise to the recent class I and II diphosphate reductases. This leaves us with the question what selective advantage was gained in that. One possibility is the uncoupling of deoxyribonucleotide production from the energy metabolism where ATP and to a lesser extent GTP have important functions. Another conceivable advantage would be an additional regulatory level that is introduced if RNRs do not produce dNTPs directly but dNDPs. While this is still only speculation, our study now opens the possibility to identify the biological processes or metabolic circumstances, under which, either deoxyribonucleoside diphosphates or triphosphates are synthesized. This will help us understand why these two different forms of deoxyribonucleotides are produced to eventually serve the DNA replication and repair processes that are so central to all known life on earth.

## Experimental procedures

### General

If not stated differently, all used chemicals were purchased from Sigma Aldrich. Ribonucleotides and deoxyribonucleotides were obtained from Jena Bioscience.

### Mutagenesis

The introduction of mutations for targeted amino acid exchange was performed by Ω-PCR with the QuikChange II Site-Directed Mutagenesis Kit (Agilent Technologies). As a template, the expression plasmids pET28b(+) were applied, containing the respective nrdJ gene between the restriction sites NdeI and HindIII (16, 17). The mutagenesis was performed according to the manufacturer's instructions from the QuikChange II Site-Directed Mutagenesis Kit. The mutagenesis product was transformed into electrocompetent *Escherichia coli* TOP10. The identity of the variant was confirmed by plasmid preparation (Miniprep kit, Macherey-Nagel GmbH & Co. KG) and sequencing (Microsynth Seqlab GmbH). Plasmids with the confirmed mutation were transformed into *E. coli* BL21(DE3) for recombinant gene expression.

### NrdJ enzyme provision

In this study, two previously described model enzymes were utilized, the NrdJm from Thermus Virus P74-23 for nucleoside triphosphate reductases and the NrdJd from *S. nassauensis* for nucleoside diphosphate reductase. The expression and purification of both enzymes was performed as described earlier (16, 17). For the so far undescribed NrdJ enzymes from *Parvibaculum* sp. and *Bacteroides* sp. UBA7333, synthetic, codon-optimized genes were purchased from Twist Bioscience in a pET28a(+) expression vector with N-terminal His-Tag (sequences are given in the supporting information). For all enzymes, a baffled shake flask with 600-ml LB medium containing 30 μg mL^−1^ kanamycin was inoculated from a preculture to an *A*_600_ of 0.1. The cultures were incubated at 37 °C and 130 RPM until reaching an *A*_600_ of 1.0.

For TVNrdJm WT and variants, expression was induced by addition of IPTG to a final concentration of 0.1 mmol L^−1^ and further incubation for 4 h at 37 °C and 130 RPM. For SnasNrdJd WT and variants, the cultures were cooled down on ice for 5 min after reaching an *A*_600_ of 1.0. Then, expression was induced by addition of IPTG to a final concentration of 0.1 mmol L^−1^ and further incubation for 18 h at 15 °C and 130 RPM. For the expression of the WT enzymes from *Parvibaculum* sp. and *Bacteroides* sp. UBA7333, the cultures were cooled down on ice for 5 min after reaching an *A*_600_ of 1.0. Then, expression was induced by addition of IPTG to a final concentration of 0.1 mmol L^−1^ and further incubation for 18 h at 15 °C and 130 RPM. In the supporting information, the purification is described and SDS-PAGE of all produced and purified enzyme variants is shown (Fig. S1).

### RNR assay

The ribonucleotide reductive activity of the WT enzymes and variants was determined by measurement of the production of deoxyribonucleotides over time *via* HPLC. The standard reaction mixture contained 2 μmol L^−1^ NrdJ enzyme, 0.5 mmol L^−1^ GTP or GDP (substrate), 0.5 mmol L^−1^ dTTP (effector), 10 μmol L^−1^ 5′-deoxyadenosylcobalamin (cofactor B12), 20 mmol L^−1^ tris(2-carboxyethyl)phosphine or DTT, 20 mmol L^−1^ MgCl_2_, and 50 mmol L^−1^ Tris, with pH = 8.0. Tris(2-carboxyethyl)phosphine was used as a reducing agent for the NrdJm from Thermus virus P74-23 while DTT was applied for the NrdJd from *S. nassauensis*. The reaction was started by addition of the enzyme and incubated for 5 to 15 min at 40 °C. The reaction was stopped by addition of one-volume-equivalent methanol, followed by an incubation at 70 °C for 10 min. The precipitated enzyme was removed by centrifugation. To the 1:1 sample:methanol mixture, four volumes of water were added and subjected to HPLC analysis. For the initial screening assays and the competition experiments (Fig. 2), the assays were performed for 2 h with the 10-fold enzyme concentration (20 μmol L^−1^).

### HPLC analysis

The quantification of the deoxyribonucleotide product was performed by reversed-phase HPLC as described before (19). The method applies tetrabutylammonium hydroxide to increase the resolution between ribonucleotides and the corresponding deoxyribonucleotides. The measurements were performed on a Knauer PLATINblue UHPLC (Knauer GmbH) or Knauer Smartline HPLC with a C18 reversed-phase column Knauer Eurospher 2 100-5 C18 and Phenomenex Luna Omega 1.6 μm C18 100 Å (Phenomenex), respectively. The separation was performed in a methanol gradient. The gradient, the precise settings, and the retention times of each analyte are described in the supporting information (Fig. S3).

### Kinetic parameter estimation

The kinetic parameters of the enzymes were determined by recording enzyme activity with respect to the substrate concentration, followed by nonlinear regression analysis. The regression analysis was performed in OriginPro 2016G by fitting the experimental data to the original Michaelis–Menten equation using the Levenberg–Marquardt algorithm. The error values in the kinetic data represent the standard error from the nonlinear regression analysis.

### Structural investigation

For the investigation of the structural determinants based on the three-dimensional structure of the enzymes, the molecular visualization tool YASARA was applied (20). Homology models of all relevant NrdJ enzymes were created *via* the SWISS-MODEL server (21, 22, 23) and compared at the relevant positions. As templates for the creation of the homology models, the crystal structures of the NrdJ from *T. maritima* (PDB ID: 1XJN) and the NrdJm from *L. leichmannii* (PDB ID: 1L1L) were applied.

### Phylogenetic distribution of motifs

We used the alignment underlying the previously published NrdJ phylogeny (Figshare: 10.17045/sthlmuni.5178415; phosphate-binding motif selection available in the Seaview file (17)) to identify different phosphate-binding motifs in NrdJs. The sequence logos were constructed with Skylign (24) using the alignment of 706 sequences with the P-N-S-P motif, 105 sequences with the P-A-G-R motif, and 58 sequences with the P-Q-G-S-P motif.

### NrdJ co-occurrence

The Genome Taxonomy Database (GTDB) ((25, 26), https://gtdb.ecogenomic.org/) is a standardized taxonomy of bacteria and archaea, based on genome phylogeny. The database includes a set of representative genomes spanning all known species of bacteria and archaea, according to the GTDB classification. The 24,059 GTDB representative genomes were downloaded and annotated with Prokka 1.14 (27). The annotated proteins were searched with NrdJ hidden Markov model profiles (Nouairia *et al*. in preparation) using HMMER 3.3 ((28), http://hmmer.org/). The NrdJ proteins were aligned with Clustal Omega 1.2 (29), and the substrate-specificity motifs were checked manually. Identification of multiple occurrences of NrdJ in genomes and other analyses were performed in the statistical programming language “R”.

## Data availability

All data used for the study are presented or cited in the article or the supporting information.

## Supporting information

This article contains supporting information (16, 17).

## Conflict of interest

The authors declare that they have no conflicts of interest with the contents of this article.
