# Supplementary material for: Structural determinants and distribution of phosphate specificity in ribonucleotide reductases
Source: J Biol Chem. 2021 Jul 24;297(2):101008. doi: 10.1016/j.jbc.2021.101008 (PMC8365446; doi:10.1016/j.jbc.2021.101008)
Supplement: Supporting information [file mmc1.pdf]

Supporting information: Structural determinants and distribution of phosphate specificity in ribonucleotide reductases.

Eugen Schell[1,3], Ghada Nouairia[2], Elisabeth Steiner[1], Niclas Weber[1], Daniel Lundin[2], Christoph Loderer[1]*

[1]Chair for Molecular Biotechnology, Institute for Microbiology, Technische Universität Dresden, Dresden, Saxony, Germany
[2]Department of Biochemistry and Biophysics, Stockholm University, Stockholm, Sweden
Present adress:
[3]Macromolecular Chemistry, Institute of Chemistry, Faculty of Natural Science II, Martin Luther University Halle-Wittenberg, Halle, Germany

*Corresponding author: Christoph Loderer
Email: christoph.loderer@tu-dresden.de
Running title: Structural determinants of phosphate specificity in RNRs

Keywords: ribonucleotide reductases, phosphate specificity, enzyme catalysis, enzyme kinetics, nucleoside/nucleotide biosynthesis, nucleoside/nucleotide metabolism, nucleic acid enzymology, site directed mutagenesis

1. Expression and purification of all used RNR variants

All enzymes applied in this study were produced by recombinant expression in *E. coli* BL21(DE3) with a pET28b(+) or pET28a(+) expression vector. The purification was performed by immobilized nickel ion affinity chromatography followed by a desalting column. Expression and purification were performed as described in earlier studies and in the methods section of the main manuscript (Loderer et al. 2017, Loderer et al. 2019). All enzymes except for double mutant SnasNrdJd S147G/P148R were expressed and purified successfully.

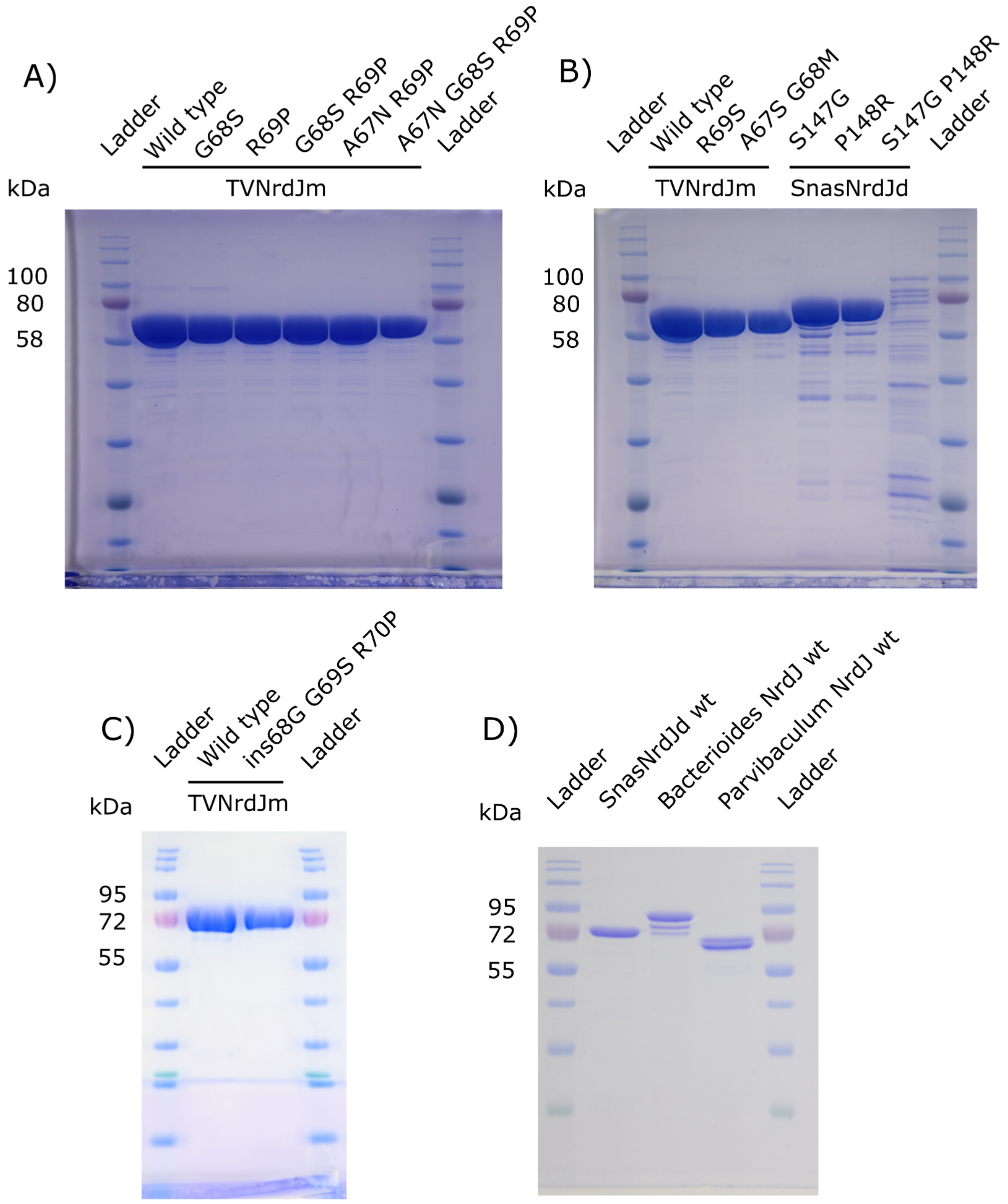

Figure S1: Purified RNRs. SDS PAGE of all the purified RNRs, used in this study. All TV74NrdJm variants and wild type enzymes are loaded in ten fold dilution and all SnasNrdJd variants are loaded in a five fold dilution. As markers, the Color Prestained Protein Standard Broad Range (11-245 kDa) (A,B) and Color Prestained Protein Standard Broad Range (10-250 kDa) (C,D) were used.

2. Parvibaculum and Bacteroides NrdJ

The NrdJ enzymes from *Parvibaculum* sp and *Bacteroides* sp. UBA7333 were selected from the available sequences with the desired motifs. The *Parvibaculum* sp. (GCA_002695905.1) and *Bacteroides* sp. UBA7333 (GCA_002471195.1) genes are derived from whole genome sequencing of the respective organism and purchased as synthetic genes from Twist Bioscience (San Francisco, USA). The genes were ordered with an *E. coli* codon optimization in the pET28a(+) expression vector inserted between the NdeI and XhoI restriction sites with terminal stop codon. This produces N-terminal fusion to the His-Tag given in the expression vector.

>NrdJ Bacteroides sp. UBA 7333 - codon optimized DNA sequence
ATGGAGAAACAGGTGTATAGTTACGAAGAAGCGTTTGAAGAATCGTTACGGTATTTCCAGGGCGACGAACTG
GCGGCGCGCGTGTGGGTGAACAAATACGCGGTGAAAGATAGTTTCGGCAATATTTACGAAAAAAGCCCCAAA
GATATGCATTGGCGTATCGCTAATGAGGTAGCGCGTATCGAAGCTAAGTACAAAAACCCGCTGTCCGCTCAAG
AACTGTTTGACTTACTGGACCACTTTAAATATATCGTGCCACAGGGGAGTCCAATGACCGGTATCGGTAACGA
CTTTCAGGTGGCCAGCCTGTCAAACTGCTTTGTCATCGGCATCGACGGTGCGGCCGATAGCTACGGTGCTAT
TATTAAGATTGATGAAGAACAGGTTCAGCTGATGAAACGTCGTGGCGGTGTGGGTCACGACCTGTCGCACAT
TCGCCCAAAGGGTTCCCCGGTTAAAAACAGCGCACTGACGAGCACGGGTCTTGTGCCGTTCATGGAACGCT
ACTCAAATAGTACACGCGAAGTCGCTCAGGATGGTCGTCGTGGTGCATTAATGCTGTCGGTTTCCATCAAACA
CCCGGACAGCGAGGCGTTTATCGATGCGAAGATGACCGAGGGAAAAGTCACAGGCGCTAACGTCAGTGTTA
AGCTGGATGATGCGTTCATGCAGGCGGCCGTGGATAAAAAACCTTATGTGCAGCGTTATCCGATTGACGCGG
AAGAGCCTACCCTGACGAAAGAAATCGATGCGGAGAACCTTTGGAAAAAGATTGTACATAACGCGTGGAAATC
CGCCGAACCAGGAGTTCTTTTCTGGGATACGATTATCCGCGAATCAGTGCCGGACTGCTATGCGGATCTGGG
TTACAAAACCGTTTCGACCAATCCGTGCGGAGAAATTCCTCTCTGCCCATACGACTCTTGTCGGCTTCTGGCA
ATCAACCTGTACAGTTACGTCATCAATCCATTCACCCCGCAGGCGAAATTTGACTTTGAATTATTTAAAAAACAT
GTGGAACTGGCACAGCGGATTATGGATGATATTATCGACCTCGAACTGGAGAAAATTGAACGTATTATCGACAA
GATTAACGCCGACCCAGAAAATGTCGAAGTCAAACGCACTGAATTGAAACTGTGGGAAAAAATTTACAAGAAA
TCCGCTCAGGGCCGCCGTACTGGTGTTGGCATTACTGCGGAAGGCGATATGCTGGCCGCCCTTGGTCTGCG
TTATGGTACCGAGGAAGCCACCGAATTCAGTGAACAGGTCCATAAAACTGTTGCGATCAACGCGTACCGTAG
CTCCGTGCAGATGGCAAAGGAACGTGGCGCGTTTGAGGTGTATAACACAGAACGTGAACAGAACAATCCATT
TATCAATCGCCTGCGTGAAGCGGATCCGCAGATGTACGAAGATATGGTCAAGTATGGGCGGCGCAACATTGC
GTGCCTGACTATTGCGCCGACCGGCACGACGAGTCTGATGACTCAAACCACTTCTGGCATCGAGCCGGTGT
TTCTCCCTGTGTACAAACGCCGTCGTAAGGTTAACCCAAACGACACGAACGTGCGGATTGATTTCGTCGATG
ATACGGGCGATGCGTTCGAGGAGTATATCGTGTTCCATCATAAATTTGTTACCTGGATGGAGGCCAATGGATAC
GACCCGTCCAAACGGTACACGCAGGAAGAGATTGACGAACTTGTGGCAAAGTCGCCATACTATAAGGCCACC
TCGAATGATGTTGATTGGCTGATGAAAGTGAAAATGCAGGGTCGCATTCAGAAATGGGTCGATCATAGCATCA
GCGTCACTATTAATTTGCCTAATGACGTGGATGAAGAACTCGTTAACCGCCTGTATGTGGAAGCTTGGAAAAG
CGGATGTAAGGGCTGTACCGTATACCGTGACGGTAGCCGCAGCGGAGTGCTTATCAGCACCAAAAGTGATAA
AAAATCTGATCTGCCGCCATGCAAACCGCCGACTGTGGTGGAAACTCGTCCGGCCGTTCTGGAAGCGGATG
TCGTCCGTTTTCAGAATAATAAAGAAAAATGGGTTGCTCTGGTCGGATTACTGGATGGTCATCCATATGAAATT
TTCACGGGGCTCCAGGATGACGATGAAGGTATTTTCATCCCAAAGACTGTCTCGATGGGCCGCATCGTCAAG
AGCTTAGATGAACATGGCAATAAACGTTATGATTTTCAATTCGAGAATAAGCGTGGCTACAAAATGACGATCGA
AGGCCTGTCCGAAAAATTCAACAAAGAATATTGGAACTATGCGAAACTTATTTCTGGCGTTCTGCGCTACCGTA
TGCCGATTGAACAAGTTATCAAATTGGTTTCGAGTCTTGAACTCGATAGCGAAAACATTAACACCTGGAAGAAC
GGCGTGGAGCGGGCGCTGAAGAAGTATATTCAGGATGGCACCGCAGCGAAAGGTAAAAAATGCCCGAATTG
CCAGAATGAGACGCTGGTGTATCAGGAGGGGTGCTTAATTTGCACTACCTGTGGGGCATCACGTTGCGGTTA
A

>NrdJ Parvibaculum - codon optimized DNA sequence
ATGAATAATCTTCTGCCGACCCCGTACCAGGAGTTTATTCATAAATCTCGTTATGCGCGTTGGATTGAAGATGA
AGGCCGCCGTGAAAATTTTGATGAGACCGTTGAGCGCTATTTAAAATTCATGGTGAACCAGGTGAAAGGGAA

GCATAACTATGACCTGTCAGGTAAAGATGTGAGCGATTTGCGCGAGGGCATTCTGAACTTGGAGATCATGCC
GAGCATGCGCGCCATGATGACGGCCGGGCCGGCTCTGGCCCGTGACAATATCTGCGGGTATAATTGCTCGTA
TATTCCGGTGGATTCCCCCGGTCCTTTGATGAATGCATGTACATCTTAATGTGTGGTACGGGGGTAGGTTTTA
GCGTAGAACGTGAAAACGTGGAAAAATTACCGACAATCAGCGACAACTTCCACGAAACGGATACGACGATTAA
GGTCGGCGACTCGAAACCCGGCTGGGCTAAGGCTTATCGTGAGTTAGTCGCCCTGCTCTATGCGGGACAGG
TTCCAGAGATTGATGTCAGTGCGGTGCGTCCGGCGGGGGAACGTCTGAAAACTATGGGGGGCCGCGCTAGT
GGGCCGCAGCCGTTAGTGGACCTTTTTAATTTCACGATCGAGACGTTTAAAAAAGCGTCGGGTCGCAAGCTT
TTCCCAATCGAGTGCCACGATCTGATGTGCAAAGTCGGAGAAATTGTGGTTGTAGGGGGTGTACGTCGTAGT
GCCCTGATTTCTCTGTCCAATCTGAACGATGATCAGATGGCGCACGCAAAATCGGGCATGTGGTGGGAAAAT
GAAGGCCAGCGCGCCCTGGCCAATAACAGCGTTTCTTACAAAGGAAAGCCTGAAATGGGTACTTTCATGCGC
GAGTGGGTGAGCCTGTACGAGTCTAAATCCGGAGAACGCGGTATCTTCAACCGTCAGGCCGCGGATATTCAA
GTTGGCCGTAACGGCCGCCGCGAACAAGGGCACATGTGGGGCACAAATCCCTGCTCCGAAATCATTTTACG
CCCCTACCAGTTTTGCAACCTTTCTGAAATTGTGGTTCGCGAATCGGACGATCTGTCGAGCCTTAAACGTAAA
GTCCGTTTGGCAACCATCTTGGGCACGCTCCAGAGCACCATGACAGATTTCAAATATCTGCGCAACGTGTGG
AAGAAAAAATACCGAAGAAGAACGGCTGCTGGGCGTTTCACTGACCGGTATTATGGACCATCCTGTGCTCAGC
AAAAATGTAGACTCGAAACGTTGGTTGGAGGAAATGAAGGAGGAAGCAGTCAAAGTAAATAAAGAGTATGCAA
AAAAACTGGGGATTCCCCAGAGCGCGGCAATTACGTGTGTCAAACCGTCAGGCACCGTGTCCCAATTAGTTG
ATGCGGCGAGTGGCATCCACGCTCGCCATCACCCGCATTACATTCGCACGGTACGCGGCGATAACAAAGACC
CGCTGACCCAATTTCTGATTGAGTCTGGCGTACATAATGAACGCGACGTTATGAAACCAGATTCTACCACGGT
CTTCTCTTTTCCGATGGAAAGCCCGAAAGGGGCAGTAACTCGCACGGAGATGACCGCGATTGAACAGCTGG
AATTATGGAAAACTTACGCACTGCACTGGTGCGAACACAAACCTAGCATTACCGTGAGTGTTAAAGAGGAAGA
GTGGATGGAGGTCGGCGCGTGGGTGTACGAAAACTTCGATGTGGCAAGCGGCGTCTCCTTTCTGCCATTTT
CCGACCATACGTATCAGCAGGCACCGTATCAAGACATTGAGCCGGATGATTATTTGGAATGGAAGCAAATGAT
GTCCCACGTGGAGATTGATTGGTCGCGTCTGACGGAGTTTGAAAAAGAAGATAACACAACTGGATCGCGTGA
ACTGGCGTGCACCGCAGGTGTCTGCGAGGTTGTTGATCTGAATGCTGCGTAA

>NrdJ Bacteroides sp. UBA 7333 - Protein sequence with N-terminal Histag
MGSSHHHHHHSSGLVPRGSH
MEKQVYSYEEAFEESLRYFQGDELAARVWVNKYAVKDSFGNIYEKSPKDM
HWRIANEVARIEAKYKNPLSAQELFDLLDHFKYIVPQGSPMTGIGNDFQV
ASLSNCFVIGIDGAADSYGAIIKIDEEQVQLMKRRGGVGHDLSHIRPKGS
PVKNSALTSTGLVPFMERYSNSTREVAQDGRRGALMLSVSIKHPDSEAFI
DAKMTEGKVTGANVSVKLDDAFMQAAVDKKPYVQRYPIDAEEPTLTKEID
AENLWKKIVHNAWKSAEPGVLFWDTIIRESVPDCYADLGYKTVSTNPCGE
IPLCPYDSCRLLAINLYSYVINPFTPQAKFDFELFKKHVELAQRIMDDII
DLELEKIERIIDKINADPENVEVKRTELKLWEKIYKKSAQGRRTGVGITA
EGDMLAALGLRYGTEEATEFSEQVHKTVAINAYRSSVQMAKERGAFEVYN
TEREQNNPFINRLREADPQMYEDMVKYGRRNIACLTIAPTGTTSLMTQTT
SGIEPVFLPVYKRRRKVNPNDTNVRIDFVDDTGDAFEEYIVFHHKFVTWM
EANGYDPSKRYTQEEIDELVAKSPYYKATSNDVDWLMKVKMQGRIQKWVD
HSISVTINLPNDVDEELVNRLYVEAWKSGCKGCTVYRDGSRSGVLISTKS
DKKSDLPPCKPPTVVETRPAVLEADVVRFQNNKEKWVALVGLLDGHPYEI
FTGLQDDDEGIFIPKTVSMGRIVKSLDEHGNKRYDFQFENKRGYKMTIEG
LSEKFNKEYWNYAKLISGVLRYRMPIEQVIKLVSSLELDSENINTWKNGV
ERALKKYIQDGTAAKGKKCPNCQNETLVYQEGCLICTTCGASRCG

>NrdJ Parvibaculum - Protein sequence with N-terminal Histag
MGSSHHHHHHSSGLVPRGSH
MNNLLPTPYQEFIHKSRYARWIEDEGRRENFDETVERYLKFMVNQVKGKH
NYDLSGKDVSDLREGILNLEIMPSMRAMMTAGPALARDNICGYNCSYIPV
DSPRSFDECMYILMCGTGVGFSVERENVEKLPTISDNFHETDTTIKVGDS
KPGWAKAYRELVALLYAGQVPEIDVSAVRPAGERLKTMGGRASGPQPLVD
LFNFTIETFKKASGRKLFPIECHDLMCKVGEIVVVGGVRRSALISLSNLN
DDQMAHAKSGMWWENEGQRALANNSVSYKGKPEMGTFMREWVSLYESKSG

ERGIFNRQAADIQVGRNGRREQGHMWGTNPCSEIILRPYQFCNLSEIVVR
ESDDLSSLKRKVRLATILGTLQSTMTDFKYLRNVWKKNTEEERLLGVSLT
GIMDHPVLSKNVDSKRWLEEMKEEAVKVNKEYAKKLGIPQSAAITCVKPS
GTVSQLVDAASGIHARHHPHYIRTVRGDNKDPLTQFLIESGVHNERDVMK
PDSTTVFSFPMESPKGAVTRTEMTAIEQLELWKTYALHWCEHKPSITVSV
KEEEWMEVGAWVYENFDVASGVSFLPFSDHTYQQAPYQDIEPDDYLEWKQ
MMSHVEIDWSRLTEFEKEDNTTGSRELACTAGVCEVVDLNAA

The activity of the enzymes was quantified in the presence and absence of the effector dTTP and the cofactor adenosylcobalamin with GTP as substrate (Figure S2). Both enzymes show conversion of GTP in the presence of both dTTP and adenosylcobalamin.

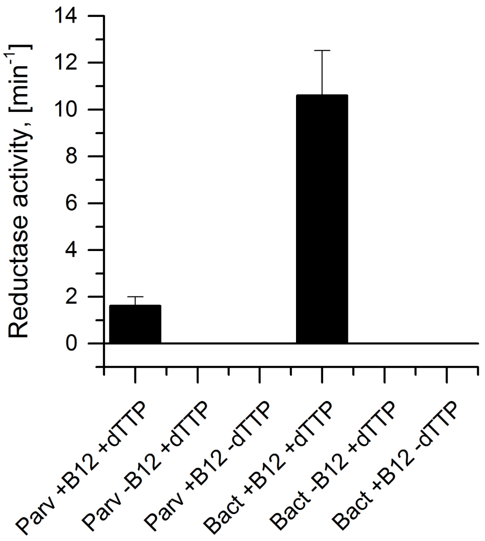

Figure S2: Activity assays with NrdJ enzymes from *Parvibaculum* sp and *Bacteroides* sp. UBA7333. The activity of the enzymes was measured in absence and presence of the effector dTTP and the cofactor adenosylcobalamin (B12) with GTP as substrate. The error bars indicate the standard deviation from three independent experiments.

3. HPLC and UHPLC Methods

Two instruments and two corresponding methods were used for the measurement of nucleotide concentrations:

- Knauer Platinblue UHPLC / C18-reversed phase column Knauer Eurosphere
  - Buffer A: 50 mM KPi (pH = 7.0), 10 mM TBAH, 10 % (v/v) methanol
  - Buffer B 50 mM KPi (pH = 7.0), 10 mM TBAH, 30 % (v/v) methanol
- Knauer Smartline HPLC / Phenomenex Luna Omega 1.6µm C18 100Å
  - Buffer A: 50 mM KPi (pH = 7.0), 10 mM TBAH, 5 % (v/v) methanol
  - Buffer B 50 mM KPi (pH = 7.0), 10 mM TBAH, 30 % (v/v) methanol

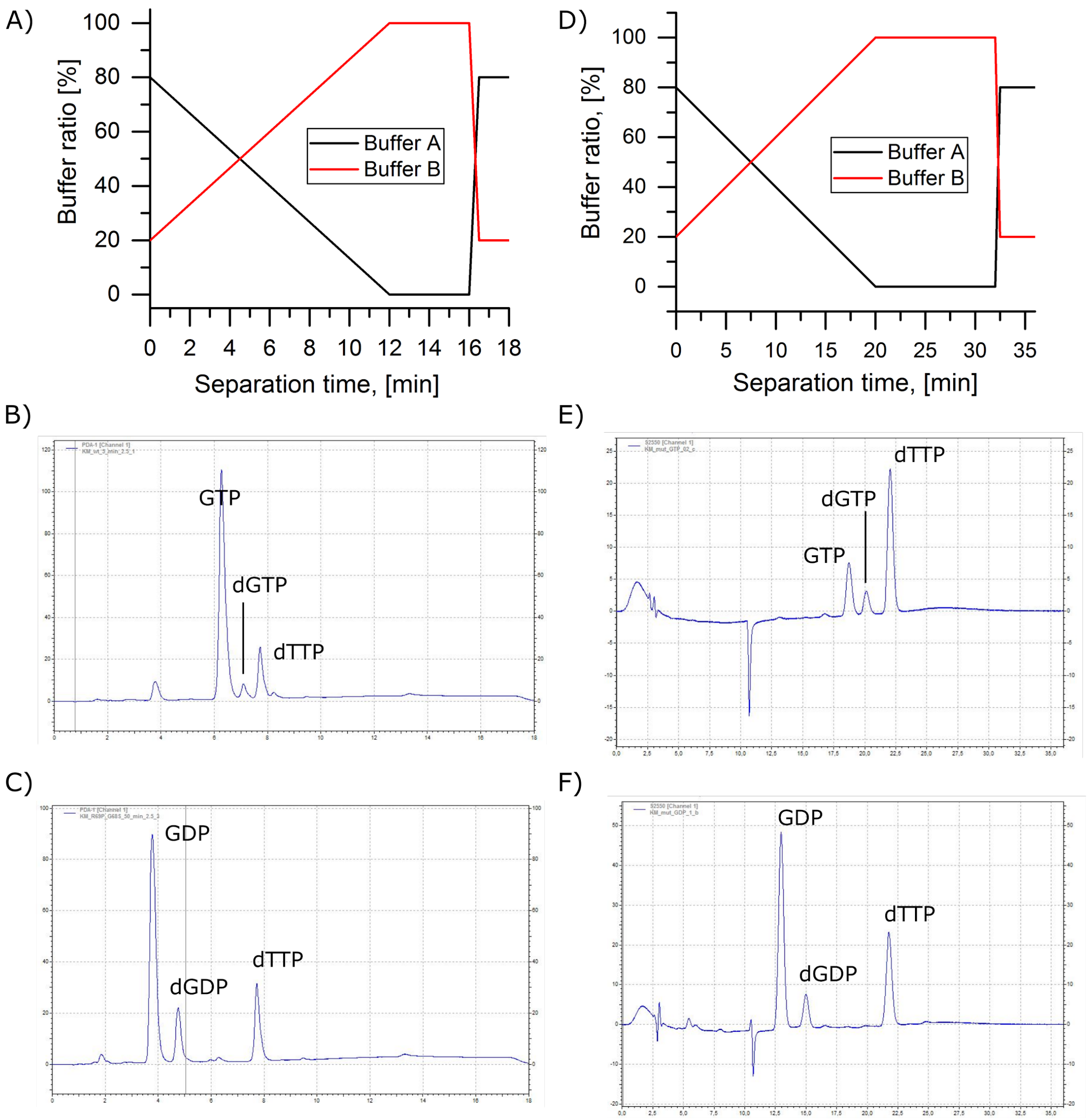

Figure S3: HPLC and UHPLC methods for enzyme activity determination. UHPLC method on the Knauer Platinblue with buffer gradient A) and exemplary chromatograms of enzymatic GTP B) and GDP C) conversions. HPLC method on the Knauer Smartline with buffer gradient D) and exemplary chromatograms of enzymatic GTP E) and GDP F) conversions.

4. Activity assays for SnasNrdJd variants S147G and P148R

The variants from SnasNrdJd were tested for activity on the substrates GTP and GDP. The reactions were performed as described in the methods section of the main manuscript with an enzyme concentration of 20 µmol L^{-1}. The incubation time of the assay was 90 min.

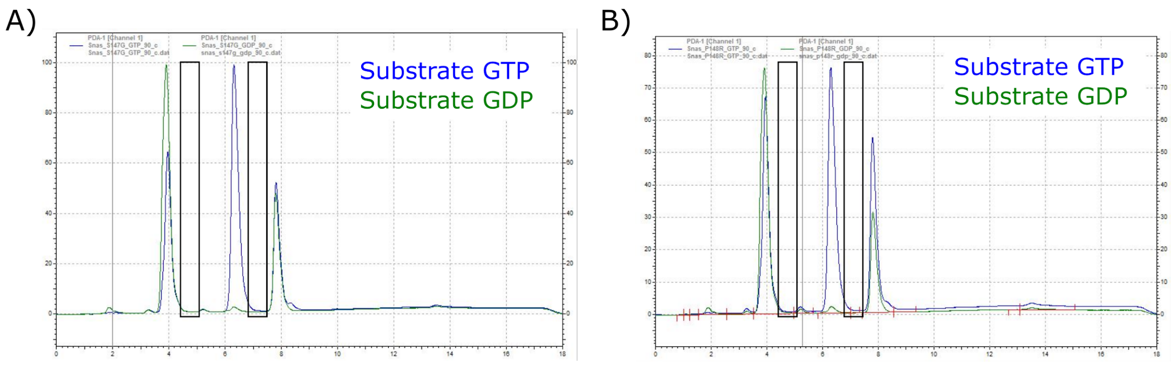

Figure S4: *Stackebrandtia nassauensis* NrdJd variant activity assays. Chromatograms of the conversion of GTP (blue) and GDP (green) by the SnasNrdJd variants A) S147G and B) P148R. The black boxes indicate the retention times where the reaction products would be expected.

5. Kinetic parameter estimation for NrdJm-G68S/R69P-double mutant

The determination of the kinetic parameters of the NrdJm-G68S/R69P-double mutant for the substrate GDP was not straightforward due to lower activities at high substrate concentrations. Data fitting with substrate surplus inhibition did not converge on parameters within the range of the experimental data. To get an approximation of the minimal K_M value, measurements were repeated in a range of substrate concentrations where the inhibition was not yet obvious. This value is a minimal value for the K_M since the application of a substrate surplus inhibition model would lead to higher K_M values.

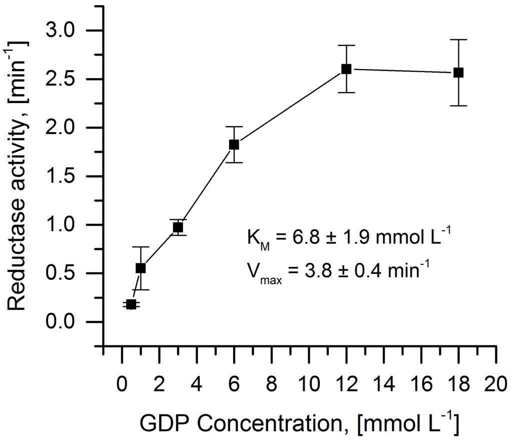

Figure S5: Kinetic parameter estimation Dependence of substrate concentration and enzymatic activity for the NrdJm-G68S/R69P-double mutant for guanosine diphosphate in the presence of the effector dTTP. The chosen concentrations are in a range where the enzyme inactivation by higher substrate concentrations is not yet obvious.

6. Activity assays for TVNrdJm variants

These variants from TVNrdJm were tested for activity on the substrates GTP and GDP. The reactions were performed as described in the methods section of the main manuscript with an enzyme concentration of 20 µmol L^{-1}. The incubation time of the assay was 60 min.

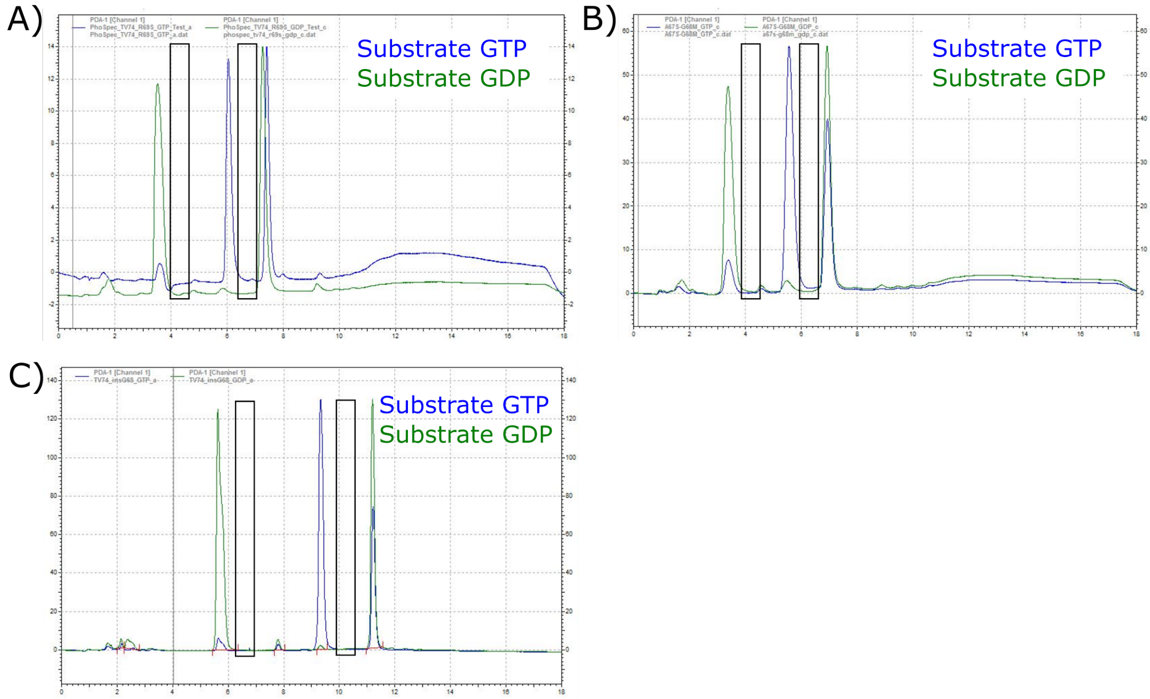

Figure S6: Thermus virus P74-23 NrdJm variant activity assays. Chromatograms of the conversion of GTP (blue) and GDP (green) by the TVNrdJd variants A) R69S, B) A67S/G68M and C) ins68G/G69S/R70P. The black boxes indicate the retention times where the reaction products would be expected.
